# Determinants and Outcome of Safe Second Trimester Medical Abortion at Jimma University Medical Center, Southwest Ethiopia

**DOI:** 10.1155/2019/4513827

**Published:** 2019-07-07

**Authors:** Yibeltal Siraneh, Ahadu Workneh

**Affiliations:** ^1^Department of Health Policy and Management, Faculty of Public Health, Institute of Health, Jimma University, Ethiopia; ^2^Department of Obstetric and Gynecology, Faculty of Medical Sciences, Institute of Health, Jimma University, Ethiopia

## Abstract

**Background:**

Although the vast majority of abortions are performed in the first trimester, still 10–15% of terminations of pregnancies have taken place in the second trimester globally. As compared to first trimester, second trimester abortions disproportionately contribute to maternal morbidity and mortality especially in low-income countries where access to safe second trimester abortion is limited. The objective of this study was to identify factors affecting and outcome of induced safe second trimester medical abortion in Jimma University medical center, Southwest Ethiopia.

**Methods:**

Institution based cross-sectional study design was used to conduct a study among women who seek safe second trimester medical abortion services and admitted at gynecology ward. All (201) eligible study subjects included were those who came for safe medical abortion service during data collection period. Data collected using pretested structured questionnaire through exit-interviewing and some clinical data abstracted from their chart. The data was entered into EpData version 3.1 then exported to SPSS version 21.0 for analysis. Variables with P-value less than 0.25 in bivariate analysis were entered into the final predictive model. Multivariable logistic regression was used to identify determinants with 95% CI and P-value < 0.05. Hosmer and Lemeshow test were used to check model fitness at P-value of 0.05. Ethical clearance was obtained and confidentiality kept using codes and patient's chart number.

**Results:**

In this study the response rate was 98.1%. Out of 201 women who participated in the study and were addmitted for safe second trimester medical abortion, 154 (76.6%) of them had complete abortion without any complication while the remaining 47 (23.4%) had incomplete abortion with one or more complication. Previous experience of abortion [AOR= 6.00, 95% CI= (3.77, 8.88)], gestational age [AOR=0.90, 95% CI= (0.07, 0.99)], parity [AOR=2.38, 95% CI= (1.04, 3.69)], cervical status [AOR=8.00, 95% CI= (5.72, 10.02)], overall waiting time for more than two weeks [AOR=0.53, 95% CI= (0.50, 0.96)], overall waiting time for two weeks [AOR=0.05, 95% CI= (0.01, 0.45)], and moderate anemia -(Hgb:7-10g/dl)-[AOR=0.07,95% CI= (0.01, 0.16)] were independent predictors for outcome of safe second trimester medical abortion.

**Conclusion:**

This finding implied that proportion of complete abortion without any complication overweighs incomplete abortions with one or more complication through induced safe second trimester medical abortion method. The outcome is strongly determined by gestational age, cervical status, previous experience of abortion, parity, moderate anemia, and overall waiting time. Induced second trimester medical abortion is already known as an effective and safe method. However, much should be done to reduce proportion of incomplete abortions by minimizing overall waiting time through intervening at low gestational age. Therefore, it is recommended that safe second trimester medical abortion services should be continued under a certain legal circumstances so as to reduce maternal morbidity and mortality.

## 1. Background

Second trimester medical abortion is termination of pregnancy between 12 and 28 weeks of gestational age. Although the vast majority of abortions are performed in the first trimester, still 10–15% of terminations of pregnancies have taken place in the second trimester period globally [1]. As compared to first trimester, second trimester abortions are disproportionately contribute for maternal morbidity and mortality especially in low-resource countries where access to safe second trimester abortion is limited [2].

Modern medical methods include induction with mifepristone and misoprostol or with misoprostol alone. The combined mifepristone regimen is significantly more effective and results in a shorter induction time than misoprostol used alone. In South Africa as well as in Ethiopia, misoprostol alone is currently the standard of care for medical termination of pregnancy in the second trimester within the public health sector [3].

Globally, abortion-related maternal deaths account 13% of which majority caused by unsafe abortions, and significant number of them occurring in the second trimester [2]. It is also a cause of maternal death that can be relatively easily reduced with the right interventions. About 210 million pregnancies occur each year throughout the world. It is estimated that 46 million of these pregnancies end in abortion: 36 million in developing countries and 10 million in developed countries [4]. 

In Africa, studies such as demonstrating the proportion of second trimester abortions are few partly because of legal restrictions. About 7% of rape cases in Brazilian women are unaware of the right to legal interruption of pregnancy after rape, so they delay in applying the procedure to get a legal abortion or at the end they try to abort in a condition that may be unsafe [5].

Evidence showed that the prevalence of induced second trimester abortion was 30% in India, 25% in South Africa, 8.6% in England and Wales, 34% in Kenya, 10% in Nigeria, and 11% in Ethiopia [6–8]. Research in Zambia revealed that determinants of the second trimester medical abortion cases at the University Teaching Hospital were socioeconomic factors (marriage, age, and income), personal factors including bynacology/obstetric related factors (parity, GA, and previous experience of abortion) and health system factors (access and quality), trauma, illness, and unknown factors. Outcomes of safe medical abortion were uncomplicated complete abortion, retained products of conception/incomplete abortion, hemorrhage, uterine perforation, pain, shock, infection, lacerations, delayed vaginal bleeding, and death with one and more than one complication [1, 9]. In Burkina Faso, three key factors were significantly associated with induced 2nd TM medical abortion such as unwanted pregnancy [OR=10.45, 95% CI=(3.59–30.41)], living in a household headed by parents [OR=6.83, 95% CI=(2.42–19.24)]; divorced or widowed [OR=3.47, 95% CI=(1.08–11.10)], being married was protective against induced abortion, with women who reported being married having 83% lower chance of having an induced abortion, and even when the pregnancy was unwanted [10].

In the case of Ethiopia, 30% of maternal deaths can be attributed to abortion and its complication. Such a high proportion of maternal deaths result from abortion (most of it being unsafe). The outcomes of these abortions are important to reinforce the need for intervention which includes uneventful evacuations, complications such as hemorrhage, incomplete abortion, shock, and even death [11]. In Ethiopia, abortion was legalized with exceptions (incest, rape, fetal defect, or when the woman's life or physical or mental health is endangered). In line with that, the standards and guidelines for reducing unsafe abortion and its consequences and how to provide the safe abortion services for those who are eligible are outlined. The termination of pregnancy act applies as far as 28 weeks of pregnancy. All cases above 12 weeks of gestation should only be managed in a unit staffed by a specialist or trained medical officer in consultation with a gynecologist as it requires providers with special training and experience [6, 12]. Although Ethiopia liberalized and revised its abortion law in 2005 with exceptions, almost 58,000 women seek care for complications of abortion in 2008 (41% had moderate or severe morbidity, such as signs of infection, which were likely related to an unsafe abortion and 7% of all women had signs of a mechanical injury or a vaginally inserted foreign body). In Ethiopia, abortion-related deaths accounted for more than 30% of maternal deaths, from which 11% was due to 2nd trimester abortion [6, 13].

There is no single reason why women have abortions in the second trimester or late seeking; much of the delay occurs prior to requesting an abortion such as women's concerns about what is involved during abortion, various aspects of women's relationships with their partners and/or parents play a role, and women's decision-making about whether to have an abortion. After requesting an abortion, delays are partly service related (waiting for appointments) and partly “woman related” (missing or cancelling appointments) [14–17].

The prevalence of second trimester abortion in Amhara region, Ethiopia, is 19.2% [18] whereas in Jimma town it is 13.7% [19] but determinants and outcome of safe second trimester medical abortion were unknown so far even if it was considered as an inherently more risky procedure [20]. Hence, the objective of this study was to assess the outcome and determinants of safe 2nd trimester medical abortion among clients admitted at gynecology ward, Jimma University Medical Center.

## 2. Methods

### 2.1. Study Setting and Design

The study was conducted in Jimma University Medical Center (JUMC), Obstetrics, and gynecology ward, comprehensive abortion (CAC) clinic, from November 1, 2016, to June 30, 2017. The center is found in Jimma town which serves a catchment population of about 15 million people. It is located 352 km southwest of Addis Ababa. The center has annual out-patient case load of 160, 000 and 45, 000 in-patients. It provides services to diverse population from three regional states: namely, Oromia, Southern Nations, Nationalities and Peoples, and Gambella. It was estimated that 180 women were admitted for safe second trimester induced medical abortion (2nd TM-MA) over a period of 6 months from previous year record. The average rate of admission for 2nd TM-MA was 30 clients per month. A facility based cross-sectional study design was used by considering all clients admitted for CAC services in Obstetrics and gynecology ward as a source population. All clients admitted for safe 2nd trimester induced medical abortion during the study period were considered as a study population. Only clients admitted for safe 2nd trimester medical abortion services without prior complication were included, whereas those who already had known medical and obstetric complications were excluded if that was an indication for admission and termination.

### 2.2. Sample Size and Sampling Technique

Sample size was calculated using a single population proportion formula, taking P = 50% (since there is no previous study conducted on outcome and determinants of safe second trimester medical abortion), n = z^2^p(1-p)/d^2^, n = 1.96^2^(0.5)(0.5)/0.05^2^, n = 384.

Assumption:

- P =Estimated proportion of complete 2nd TM-MA without any complication (50%)
- d = Marginal error/degree of precision =5% (0.05)
- *α* = Critical value at 95% CI of certainty (1.96)
- Z= Reliability coefficient
- n= Sample size estimation of single population proportion

The total estimated source population from the previous record is 180 that was less than 10,000. Hence, finite population correction formulae were used to adjust the sample size.

(1) $$\begin{array}{c} Nf=\left(\;\frac{n}{1+n/N}\;\right)=\frac{384}{1}+\frac{384}{180}=123 \end{array}$$

- where N_f_ = the sample size from a finite population
- N = number of total source population
- n = sample size estimation of single population proportion
- Finally by adding, expected NR rate = 10% of Nf (123) = 13, n = 136.

The required sample size was* 136* but all eligible subjects included considering the sample size to be more than 136 to maximize statistical power. A consecutive sampling technique was employed to include all eligible subjects who came to JUMC for safe second TM-MA services during the study period.

### 2.3. Study Variables and Operational Definitions

The dependent variable is the outcome of safe second TM-MA and independent variables are categorized under sociodemographic characteristics (age, religious affiliation-to see religious based attitude towards abortion, ethnicity, marital status, educational status, occupational status, place of residence, and own monthly income), services and perception related variables (distance from facility, service availability, referral system, transportation cost, services cost, perception towards physician skill and abortion, waiting time, and satisfaction), and obstetrics and gynecology related variables (history and type of contraceptive use, menses status, previous experience of abortion, reason for delayed termination, gestational age, gravidity, parity, status of anemia, cervical status, procedure type, and expulsion time). Important variables are operationalized as follows:

1. Outcome of safe second trimester medical abortion categorized as follows;
  - Complete abortion (uncomplicated) means expulsion of foetus in a reasonable period of time (after start of regimen within 48-72 hours, using the protocol developed by Jimma University Medical Center) without any complication.
  - Incomplete abortion (complicated) means presence of short term complication (retained products of conception/incomplete abortion, anemia, cervical/uterine/abdominal injury, shock, infection, vaginal wall lacerations, need of transfusion, and death),* and/or* failure of expulsion that leads to active surgical intervention.
  - Safe abortion means the termination of pregnancy carried out by accredited health professional with the skills or training to perform the procedure safely, in a place that meets minimum medical standard, in this case at specialized teaching medical center. However, women are able to access abortion services in specific circumstances that will be determined based on the chief complain of woman and the physician considering the legal conditions.
2. Cervical status is measured by effacement, dilatation, consistency, and its position to decide whether it is closed or open.
3. Status of anemia categorized according to the following cut off points. Such as severe anemia (Hgb<7 g/dl), moderate anemia (Hgb:7-10 g/dl), mild anemia (Hgb: 10.1-10.4 g/dl), and no anemia (Hgb >= 10.5 g/dl).
4. Short-term complication(s) is(are) that complication(s) may occur starting from the intervention until discharge from the hospital.
5. Perceived physician skill: when the service provider has both adequate working knowledge and skill to provide the expected services that measured by patient perspective. It was measured by using 5-point likert scale ranging from 1-no skill to 5-had excellent skill and the mean score used to categorize as “have no skill” = scored below mean score of 3.42 and “have good skill” = scored the mean value of 3.42 and above.
6. Attitude towards abortion was measured by using 5-point likert scale and categorized into two as they had positive attitude if scored the mean value or above, or negative attitude if scored below the mean score.
7. Satisfaction on overall waiting time indicates level of satisfaction of the client about the time that spent in the registration room, at the waiting area, at admission process, to get the physcian, consultation time, and time to start the regimen. Level of satisfaction with over all waiting time from entry to exit was measured by 5-point likert scales ranging from 1-strongly disatisfied to 5-strongly satisfied then dichotomized into two using the mean score (1.78) as a cut off point.
8. Overall satisfaction on the comprehensive abortion care given to clients measured by 5-point likert scales ranging from 1-strongly disatisfied to 5-strongly satisfied, then dichotomized into two using the mean score as a cut off point (scored below the mean-7.21-disatisfied and scored mean value and above-7.21-satisfied)

### 2.4. Method of Data Collection

Interviewer-adiminstered structured questionnaire was developed after reviewing relevant literature. A two-day training was provided for all data collectors and supervisors prior to actual data collection. Translated, pretested (5%), interviewer administered, and structured questionnaire was used to interview women at exit or during discharging process. Midwife nurses conducted exit interviews. Obstetrics and gynecology residents completed the clinical or technical part of the questionnaire from the client's chart under the supervision of the principal investigator. All questionnaires were reviewed and checked on daily bases by supervisors to assure quality of data and its completeness. Clinical data were completed by reviewing client's chart. Following the participants received the service as per the clinical standard, their respective clinical findings were recorded from their chart such as gestational age, gravidity, parity, status of anemia, cervical status, procedure type, expulsion time, retained products of conception/incomplete abortion, hemoglobin level, cervical/uterine/abdominal injury, shock, infection, vaginal wall lacerations, and need of transfusion.

### 2.5. Data Processing and Analysis

Data were checked for completeness, consistency and entered into EpData version 3.1. SPSS version 21.0 was used for statistical analysis including cleaning. A logistic regression model was used to identify explanatory variables and to control for confounding variables. Candidate variables at p- value<0.25, in bivariate analysis, were entered into multivariable logistic regression. Binary logistic regression analysis was used to see the values of COR which was declared as significant at p-value < 0.05. Backward model selection method was used. The degree of association between dependent and independent variables was assessed using an adjusted OR with 95% CI at p-value < 0.05. The Hosmer and Lemeshow test were used to check model fitness at P-value of 0.05.

## 3. Results

### 3.1. Socio-demographic Characterstics

Out of 205 eligible study subjects, the response was 201 (98.1%). The average age of participants was 21.26 (16.43-26.09) year. Nearly half 85 (42.3%) of them were between the ages of 15-19 years. Nearly half (86) (42.8%) were followers of Orthodox and Muslim with equal percentages. More than half 103 (51.2) were of Oromo ethnicity. More than three-fourths 170 (84.6%) were single in marital status and 168 (83.6%) literate. Majority (111) (55.2%) of them were student in their occupational status. More than two-thirds 139 (69.2%) were urban residents and more than three-fourths 159 (79.1%) had a monthly individual income of 0 to 22.22USD ranging from 0 to 133.33 USD (refer to Table 1).

### 3.2. Obstetric and Gynecology History

Almost two-thirds of women 131 (65.2%) had no history of using contraceptives. Among the users 70 (34.8%), more than half 37 (52.9%) used IUCD. Nearly three-fourths of the women's menses status were regular 143 (71.1%). One-fourth (26) (12.9%) of women had previous experience of abortion. Among women who had experience of abortion almost all of them were nduced 21 (80.8%). Out of 26 (12.9%) women, 18 (69.2%) of them aborted once and the remaining aborted more than once. Majority 15 (57.7%) of them aborted at first trimester. Type of induction employed previously for 21 (80.8%) of them was safe medical abortion only. Ten (5%) of them interved by themselves but failed to terminate the last pregnancy using modern drug by own. More than one-third 75 (37.3%) of them replied as the only reason for delay (>=3month) was fear of stigma which is followed by fear of cost 35 (17.4%). Near to half (91) (45.3%) of them had decided termination by own which is followed by one of the family/relatives (87) (43.3%) (refer to Table 2).

### 3.3. Obstetric and Outcome of Safe 2nd TM-MA

Out of all women participated, the majority 100 (59.8%) were between 12-18 weeks of gestational age. Three-fourths of the women were gravida one. Hemoglobin (Hgb) level at admission showed that almost all of them were non anemic. For 179 (89.1%) women amount of bleeding estimated as it was within in the normal range (expected). None of the women had pelvic infection at admission. More than three-fourths 154 (76.6%) of the women had complete expulsion using medication only. For 62 (40.8%) women the total time taken to expel using recommended dose of medication was 72 hours or less (refer to Table 3).

### 3.4. Abortion Services and Perception towards Abortion

For the majority of women 120 (59.7%) heath facility took less than 30 minutes to reach without transportation.More than half 112 (55.7%) responded that no abortion services provided at nearby health facility. Nearly all 183 (91%) participants were self-referred clients. More than three-fourths 159 (79.1%) of them did not face transportation problem. Almost half 98 (48.8%) of them replied that abortion service was free of cost and nearly all 193 (96.0%) replied that the service providers considered their interest. Nearly half 91 (45.3%) of participants rated or percieved physician as had very good skills (refer to Table 4).

More than one-third 74 (36.8%) of clients were agreed that terminating pregnancy is immoral. However, 81 (40.3%) of them strongly agreed about terminating pregnancy for acceptable reason is okey. More than two-thirds 139 (69.2%)of them had a positive attitude towards abortion. Majority 127 (63.2%) of women were satisfied with the overall services provided. One hundred twenty (59.7%) women replied as the time spent to get abortion services was very long. Around half 97 (48.3%) of them replied that hospital registration time was relatively long. However, majority 122 (60.7%) of women were satisfied with overall waiting time from entry to exit. Almost three-fourths 145 (72.1%) of them were satisfied with overall services. Almost all 181 (90%) of them stayed for one week at hospital, and 48 women got the service after waiting for more than 2 weeks. After registration, 139 (69.2%) women replied that their waiting time to see the physician for their appointment was 30 minutes or less (refer to Table 4).

### 3.5. Determinants and Outcome of Safe 2nd TM-MA

The bivariate logistic regression model consisting of variables (age, religion, ethnicity, marital status, educational status, occupational status, place of residence, own monthly income, history of using contraceptive, perceived cause of pregnancy, menses status, previous experience of abortion, gestational age, gravidity, parity, hemoglobin (Hgb) level at admission, cervical status, amount of bleeding estimated, procedure type employed to complete expulsion, duration of hospital stay, and overall waiting time) with dependent variable were tested one by one to identify candidates using p-value of <0.25. Among these variables only marital status, educational status, previous experience of abortion, gestational age, parity, cervical status, and overall waiting time and hemoglobin (Hgb) value were selected as a candidate for multivariable logistic regression analysis. Out of 201 women, more than three-fourth 154 (76.6%) of them had completed abortion without any complication while the remaining 47 women end up with incomplete abortion with one or more complication. From the final model, variables found to be significantly associated with the outcome of safe second trimester medical abortion were contraceptive use, previous experience of abortion, gestational age, parity, cervical status, overall waiting time, and hemoglobin (Hgb) value (refer to Table 5).

The odds of complete abortion without any complication among women with previous experience of abortion was 6 times higher as compared with those who had no experience of abortion [AOR = 6.001, 95% CI = (3.766, 8.885)]. The odds of complete abortion without any complication decreased as gestational age increased which means the odds of complete abortion without any complication among women with gestational age between 24.1-28 weeks was 0.9 times lower as compared to those between 12 and 18 weeks [AOR = 0.902, 95% CI = (0.074, 0.986)]. The odds of complete abortion without any complication among multiparas women was 2.4 times higher as compared to nulliparous [AOR = 2.384, 95% CI = (1.040, 3.693)]. Similarly, the odds of complete abortion without any complication among women with open cervical status before taking recommended medication was 8 times higher as compared to those who had closed or unchanged cervical status [AOR = 8.001, 95% CI = (5.715, 10.015)]. When overall waiting time increase, the probability of complete abortion without any complication will decrease. The odds of complete abortion without any complication among women who waited for more than two weeks to receive abortion services was 0.5 times lower as compared to those waited for one week [AOR = 0. 531, 95% CI = (0.504, 0.963)]. Similarly, the odds of complete abortion without any complication among women with moderate anemia (Hgb:7-10 g/dl) was 0.07 times lower as compared to those with no anemia [AOR = 0.071, 95% CI = (0.004, 0.163)] (refer to Table 5).

## 4. Discussion

In Ethiopia, misoprostol alone is currently the standard of care for safe medical termination of pregnancy in the second trimester. Out of all women participated in our study, more than three-fourth of them had complete abortion without any complication while less than one fourth had incomplete abortion with one or more complication, after they were admitted for induced safe medical abortion during second trimester period. The short-term outcome of safe second trimester medical abortion was uncomplicated complete abortion (expelled using medication only without any complication), and failure of expulsion that needed active surgical intervention (dilatation and curate), retained products of conception that needed manual vacuum aspiration (MVA), and anemia (mild to moderate) were considered as incomplete abortion with complication. But no record of injury or perforation or lacerations due to the procedure on cervix or uterus or bowel or bladder or vagina, shock, infection, delayed vaginal bleeding, blood transfusion, fever, diarrhea, and death.

Result of our study is supported by other research conducted elsewhere that revealed as uncomplicated complete abortion, retained products of conception/incomplete abortion, hemorrhage, uterine perforation, shock, infection, lacerations, delayed vaginal bleeding, and death were outcomes with 68 (46.9%) had one, 47 (32%) had two and 22 (15.2%) had three complications [1]. However, those who had unfavorable outcome in particular retained products of conception and shock accounted 16 (11%) which is lower than our study result. This may be due to sensitivity of measurements applied in our case-refer operational definition given to the dichotomized variables [1, 3]. A research study conducted on outcome of safe 2nd TM-MA in Singapore revealed that there was a high incidence of minor side-effects such as fever (80%) and diarrhea (13%) with low incidence of major complications such as blood transfusion (0.9%) and readmission (0.2%). In our study unfavorable outcomes were almost comparable with other research findings in which 26 patients (27.3%) required evacuation of their uterus (MVA or curate) to complete the abortion [3]. The outcome differences between this study and ours' may be due to women's sociodemographic characterstics, retrospective nature of that study analyzed from secondary data, which excluded women above 24 weeks of gestational age and study setting. Our study also revealed less number of women who had incomplete abortion with one or more complication as compared to the national level report [11].

In our study, more than one-third of them replied as the only reason for delay (>=3month) was fear of stigma whearas the most frequent reason was conflict with partner and not informed about abortion services [1, 16, 17]. Such findings imply that study partcipants lack awarness about abortion services available including as it is charge-free. Our study implied that there is no single reason why women have abortion in the second trimester which is supported by an other study. Much of the delay occurs prior to requesting an abortion and relates to women's perception towards abortion, various aspects of women's relationships with their partners, and women's decision-making about whether to have an abortion; after requesting an abortion, delays are service related (waiting for appointments) [14].

Out of all women who participated in our study, majority were between 12 and 18 weeks of gestational age who had more favorable outcome as compared to other findings. The difference may be due to three-fourth of the women were gravida one and the rest were nulliparous as compared with other studies. More than three-fourth of women had complete abortion without any complication through medication only which is much higher than an other study revealed 16 (11%) [1]. The difference may be due to the inclusion criteria in which the other study included those women who had prior medical or obstetric complications whearas in our case we included only women admitted and indicated to safe medical abortion. For 62 (40.8%) women the total time taken to expel using recommended dose of medication only was 72 hours which is consistent with the medical center protocol. Time in hours between commencement of bleeding and expulsion of fetus varied from women to women. The maximum time was 96 hours and the minimum was 12 hours. In this study, there appeared to be a lower evacuation rate by MVA if the termination was carried out in early gestation (12.1-18 weeks).This fits with the traditional belief that morbidity due to termination is lower if it is carried out as early as possible. One possible reason may be due to a higher response to the misoprostol at early gestational age with the development of its receptors. This finding is contrary to findings from national university hospital in Singapore. The exact reason for this observation or difference is unknown [3]. Almost near to all of our study participants stayed for one week at hospital and 93 (46.3%) of them got the sevice after waiting for 1 week, and 48 women got the service after waiting for more than 2 weeks. This is totally incompatable with WHO guidelines in which a woman who is eligible for pregnancy termination should obtain the service within three working days. This time is used for counseling and diagnostic measures necessary for the procedure [2].

According to our study, previous experience of abortion, gestational age, parity, cervical status, overall waiting time for more than two weeks, overall waiting time for two weeks, and moderate anemia were independent predictors of outcome of safe second trimester medical abortion which is not supported by a study conducted in Singapore showing no significant difference in treatment outcomes when taking maternal characteristics into consideration (parity, race, marital status, previous deliveries, and gestational age). However, age and gestational age were significant predictors in similar way [3]. This may be justified as both are cross-sectional study designs that can establish temporary factors which may change or vary over time in different settings.

The likelihood of having complete abortion without any complication was 6 times higher among women who had previous experience of abortion as compared with those women who had no experience of abortion. This may be due to the relationship with number of parity or deliveries because multiparas women were 2.4 times more likely to have this good outcome as compared to nulliparous which is supported by an other study [3]. And the probability of developing favorable outcome increased when overall wating time decreased. Similarly, women with open cervical status before taking recommended medication were 8 times more likely to have favorable outcome as compared to women who had closed or unchanged cervical status. This implied that cervical position, consistency, and effacement were vital in the process of complete abortion. The methods of uterine evacuation varied from women to women but the overall outcome of the patient was not significantly affected by the procedure type employed.

The possible limitation of this study was the clinical part of data abstracted from the secondary data or patient's chart. This finding may be biased by the physician's knowledge and skill who followed and did the procedures as well as documenting reliable information on the chart. Some of the items were perception related and self-reported. Social desirablity bias and interviewer bias might be also an other potential biases for such study condcuted on sensetive issues(abortion). This finding may not be generalized to the target population because of nonprobablity sampling technique used at a single facility.

## 5. Conclusion and Recommendation

In conclusion, more than three-fourth of women had complete abortion without any complication while the remaining one-fourth had incomplete abortion with one or more complications.

Previous experience of abortion, gestational age, parity, cervical status, overall waiting time, and moderate anemia were independent predictors of outcome of safe second trimester medical abortion. Therefore, we recommend that safe second trimester medical abortion services should be continued under certain conditions as per the national legislation so as to reduce maternal morbidity and mortality. Induced safe second trimester medical abortion is already known as an effective and safe method for midtrimester pregnancy termination. However, much should be done to reduce proportion of incomplete abortion with complication and overall waiting time to improve patients' satisfaction and outcome of abortion. Single protocol is used in Ethiopia to guide medical abortion and some women are at high risk of facing incomplete abortion with some forms of complication(s). Hence, we recommend that the Ministry of Health should look for additional or optional protocol to practice the medical abortion according to the women's characteristic using treatment algorithm.

In Ethiopia, for women who have unintended pregnancies, substantial effort needs to be made to ensure safe and effective termination methods are available for women who choose this (medical abortion) option in a condition where it is legal. Women who are eligible for pregnancy termination should have the necessary information to seek abortion care as early in pregnancy as possible. Health professionals should inform women as comprehensive abortion services are free of charges, and to reduce stigma since those are the major reason for delay.

## Figures and Tables

**Table 1:** Sociodemographic characteristics of women who terminated their pregnancy using safe medical abortion at 2nd trimester in JUMC from November 1, 2016 to June 30, 2017.

Variable	Variable category	Frequency	Percent (%)
Age in years	15-19	85	42.3
	20-24	68	33.8
	25-29	31	3.5
	30-34	10	5.0
	35 and above	7	15.4

Religious affiliation	Orthodox	86	42.8
	Muslim	86	42.8
	Protestant	27	13.4
	Others*∗*	2	1.0

Ethnicity	Oromo	103	51.2
	Kaffa	42	20.9
	Dawuro	20	10.0
	Amhara	31	15.4
	Other*∗∗*	5	2.5

Marital status	Single	170	84.6
	Married	8	4.0
	Divorced	12	6.0
	Widowed	11	5.5

Educational status	Unable to read and write	33	16.42
	Completed Grade 1-8	11	5.46
	Completed Grade 9-10	34	16.92
	Completed Grade 11-12	54	26.87
	Completed Grade 12 or +	69	34.33

Occupational Status	House wife	19	9.5
	Merchant	18	9.0
	Student	111	55.2
	Government employee	17	8.5
	Daily laborer	24	11.9
	Other*∗∗∗*	12	6.0

Place of residence	Urban	139	69.2
	Rural	62	30.8

Own monthly income (in birr), 1USD = 22.5ETB	0-500	159	79.1
	501-1000	25	12.4
	1001-1500	9	4.5
	Above 1500	8	4.0

Other*∗*-Catholic and Adventist, Other*∗∗*-Tigrie and Guragie, and Other*∗∗∗*-Private Employee and Farmer.

**Table 2:** Obstetric history of women who terminated their pregnancy using a safe medical abortion in their 2nd trimester in JUMC from November 1, 2016, to June 30, 2017.

Obstetric History	Variable category	Frequency	Percent (%)
History of ever using contraceptive	Yes	70	34.8
	No	131	65.2

Type of contraceptive used in past	Injectable	4	5.7
	Pills	14	20.0
	Implants	15	21.4
	IUCD	37	52.9

Menses status	Regular	143	71.1
	Irregular	58	28.9

Previous history of abortion	Yes	26	12.9
	No	175	87.1

Type of Previously experienced abortion	Induced	21	80.8
	Spontaneous	5	19.2

Frequency of termination Previously	Once	18	69.2
	twice	7	26.9
	Thrice or more	1	3.8

Previously experienced abortion period	First trimester	15	57.7
	Second trimester	11	42.3

Type of induction employed previously	Safe medical abortion	21	80.8
	Surgical abortion	5	19.2

Type of intervention failed to terminate the last pregnancy	Not intervened	183	91.04
	Modern drug by own	10	4.98
	Traditional medicine	6	2.99
	Instrumentally by traditionalist	2	0.99

Self-reported reason for delay in seeking abortion (>=3month)	Fear of stigma	75	37.31
	Afraid of hospital workers	5	2.49
	Fear of cost	35	17.41
	Fear of legal issue	8	3.98
	Unaware of pregnancy	31	15.42
	Family members' pressure delayed decision to seek medical help	3	1.49
	Did not have information that the hospital could terminate	4	1.99
	Delayed in getting a clinic appointment	10	4.98
	Feared being arrested ( I don't know the legal aspect of abortion)	3	1.49
	Feared the effects of abortion on my health	9	4.48
	Tried other methods of abortion but failed	18	8.96

Who decided to terminate the current pregnancy	Sexual Partner	18	9.0
	You only	91	45.3
	You and sexual partner	5	2.5
	One of the family/relatives	87	43.3

**Table 3:** Obstetric and outcome of abortion related variables of women terminated pregnancy using safe medical abortion at 2nd trimester in JUMC from November 1, 2016, to June 30, 2017.

Obstetric and outcome of abortion	Variable category	Frequency	Percent (%)
Gestational Age in weeks	12-18-Late/Delayed	100	49.8
	18.1-24- late of late	88	43.8
	24.1-28-Extremely late	13	6.5

Gravidity	Gravida I	151	75.1
	Gravida II-III	41	20.4
	Gravida IV or above	9	4.5

Parity	Nulliparous	169	84.0
	Primiparas	18	9.0
	Multiparas	14	7.0

Hemoglobin (Hgb) level at admission	Severe Anemia (Hgb<7 g/dl)	0	0.0
	Moderate Anemia (Hgb:7-10 g/dl)	3	1.5
	Mild Anemia (Hgb: 10.1-10.4 g/dl)	2	1.0
	No Anemia (Hgb >=10.5 g/dl)	196	97.5

Cervical status	Closed	46	22.9
	Open	155	77.1

Amount of bleeding estimated	Expected	179	89.1
	More than expected	16	8.0
	Not documented	6	3.0

Status of pelvic infection at admission	Absent/Not Diagnosed	201	100.0
	Present/Diagnosed	0	0.0

Procedure type employed to complete expulsion	MVA	36	17.9
	E and C (Curate)	13	6.5
	Medical abortion only	152	75.6

Outcome of medical abortion after taking recommended regimen	Expelled using medication only without any complication	154	76.6
	Due to failure of expulsion, active surgical intervention (D and C) was done	4	2.0
	Retained products of conception/ incomplete abortion done by MVA	39	19.4
	Anemia	4	2.0
	Other (Injury, Shock, Infection, Need of transfusion, fever, diarrhea and Death)	0	0.0

Total time taken to expel using recommended dose of medication only	12 Hours	1	0.7
	14 Hours	2	1.3
	24 Hours	4	2.6
	36 Hours	37	24.3
	48 Hours	43	28.3
	72 Hours	62	40.8
	96 Hours	3	2.0

**Table 4:** Health services and perception related variables of women who terminated their pregnancy using safe medical abortion at the 2nd trimester in JUMC from November 1, 2016, to June 30, 2017.

Health services & perception variables	Variable category	Frequency	Percent (%)
Distance from clients' home to any heath facility	Less than 30 minutes	120	59.7
	30' to 1Hr	40	19.9
	More than 1 Hr	41	20.4

Does the facility give abortion services?	Yes	83	41.3
	No	112	55.7
	Am not sure	6	3.0

Referral type	Self-referred	183	91.0
	Formally Referred	18	9.0

Transportation problem to reach JUMC	Yes	42	20.9
	No	159	79.1

Transportation cost	Cheap	82	40.8
	Expensive	36	17.9
	Fair	83	41.3
	No cost/Foot	0	0

Abortion service cost	Free	98	48.8
	Expensive	19	9.5
	Cheap	84	41.8

Perceived physician skill	No skill	11	5.5
	Fair skill	37	18.4
	Good skill	36	17.9
	Very good skill	91	45.3
	Excellent Skill	26	12.9

Terminating pregnancy is immoral	Strongly disagree	17	8.5
	Disagree	71	35.3
	Neutral	11	5.5
	Agree	74	36.8
	Strongly agree	28	13.9

Support of terminating pregnancy for acceptable reason	Strongly disagree	28	13.9
	Disagree	15	7.5
	Neutral	0	0
	Agree	77	38.3
	Strongly agree	81	40.3

Level of satisfaction with the overall services/care provided	Strongly dissatisfied	16	8.0
	Dissatisfied	29	14.4
	Uncertain	1	.5
	Satisfied	127	63.2
	Strongly satisfied	28	13.9

The time spent to get abortion services	long	120	59.7
	Short	39	19.4
	Appropriate	42	20.9

Which time was very long relatively (which part took the longest time)?	Registration	97	48.3
	Wait to see physician	46	22.9
	Consultation	58	28.9

Level of satisfaction with over all waiting time from entry to exit	Strongly dissatisfied	16	8.0
	Dissatisfied	31	15.4
	Uncertain	1	.5
	Satisfied	122	60.7
	Strongly satisfied	31	15.4

Duration of hospital stay	One week	181	90.0
	two weeks	20	10.0
	More than two weeks	0	0.0.

Overall waiting time to get the service with appointment	Waiting for 1 week	93	46.3
	Waiting for 2 weeks	60	29.9
	Waiting for >2wks	48	23.9

Waiting time to see the physician on the date of appointment	30 minutes and less	139	69.2
	30 minutes to 1hour	27	13.4
	1hour to 3 hours	15	7.5
	3 hours to 6 hours	8	4.0
	6 hours to half day	2	1.0
	One day and above	10	5.0

**Table 5:** Bivariate and multivariate logistic regression analysis of factors affecting outcome of safe 2nd trimester medical abortion among women terminated pregnancy in JUMC November 1, 2016 to June 30, 2017.

Variable with category		Outcome of safe 2nd TM-MA		Crude OR (95% CI)	AOR (95% CI)
		Uncomplicated F (%)	Complicated F (%)		
Marital status	Single	135 (87.7)	35 (74.5)	1	1
	Married	5 (3.2)	3 (6.4)	3.214(1.927,6.148)*∗*	2.566 (0.696, 5.458)
	Divorced	8 (5.2)	4 (8.5)	1.569(0.285, 8.621)	1.274(0.195, 8.346)
	Widowed	6 (3.9)	5 (10.6)	1.274(0.195, 8.346)	1.569(0.285, 8.621)

Educational status	Unable to read and write	21 (13.6)	12 (25.5)	0.461(0.207,1.026)	0.588 (0.247, 1.398)
	Able to read and write	133 (86.4)	35 (74.5)	1	1

Contraceptive use	Yes	48 (31.2)	22 (46.8)	0.515(0.264,1.802)	0.304 (0.064, 1.641)
	No	106 (68.8)	25 (53.2)	1	1

Previous experience of abortion	Yes	11 (7.1)	15 (31.9)	7.012 (5.234, 9.021)*∗*	6.001 (3.766, 8.885)*∗*
	No	143 (92.9)	32 (68.1)	1	1

Gestational age	12-18 wks	78 (50.6)	22 (46.8)	1	1
	18.1-24 wks	65 (42.2)	23 (48.9)	2.043(0.199,3.481)	1.160 (0.328, 4.101)
	24.1-28 wks	11 (7.1)	2 (4.3)	1.574(1.195, 8.346)*∗*	0.902 (0.074, 0.986)*∗*

Parity	Nulliparous	127 (82.5)	42 (89.4)	1	1
	Primiparas	16 (10.4)	2 (4.3)	2.012(1.202,12.456)	1.720(0.189, 13.692)
	Multiparas	11 (7.1)	3 (6.4)	3.213 (1.502,5.654)*∗*	2.384 (1.040, 3.693) *∗*

Cervical status	Closed	6 (3.9)	40 (85.1)	1	1
	Open	148 (96.1)	7 (14.9)	10.011(7.213,12.011)*∗∗*	8.001 (5.715, 10.015)*∗∗*

Overall waiting time	Waiting for 1 week	69 (44.8)	24 (51.1)	1	1
	Waiting for 2weeks	39 (25.3)	21 (44.7)	0.096 (0.020, 0.461)*∗∗*	0.054 (0.006, 0.453)*∗∗*
	Waiting for >2 wks	46 (29.9)	2 (4.3)	0.849 (0.365, 1.536)*∗∗*	0. 531 (0.504, 0.963)*∗∗*

Hemoglobin (Hgb) value	Moderate Anemia (Hgb:7-10 g/dl)	1 (0.6)	2 (4.3)	0.102 (0.012,1.001)*∗*	0.071 (0.004, 0.163)*∗*
	Mild Anemia (Hgb: 10.1-10.4 g/dl)	0 (0.0)	2 (4.3)	0.502 (0.101, 1.232)	0.101 (0.006, 1.682)
	No Anemia (Hgb >=10.5 g/dl)	153 (99.4)	43 (91.5)	1	1

*Key:*  *∗* P-value <0.05, *∗∗* P-value <0.001, COR-Crude odd ratio, AOR-Adjusted odd ratio, F-Frequency

## Data Availability

The datasets used and/or analyzed during the current study are available from the corresponding author on reasonable request. The SPSS (software) which is completed with raw dataset can be also shared. All data generated or analyzed during this study are included in this manuscript in the annex part and as supplementary information using tables.

## Abbreviations

CAC: Comprehensive abortion care
CI: Confidence interval
CIRHT: Center for International Reproductive Health Training
GA: Gestational age
IUCD: Intrauterine contraceptive device
JUIH-IRB: Jimma University Institute of Health–institutional review board
JUMC: Jimma University Medical Center
MoH: Ministry of Health
MVA: Manual Vacuum Aspiration
OR: Odds Ratio
Safe 2nd TM-MA: Safe second trimester medical abortion
SD: Standard deviation
SPSS: Software package for social sciences
WHO: World Health Organization.
